# States with *higher* minimum wages have *lower* STI rates among women: Results of an ecological study of 66 US metropolitan areas, 2003-2015

**DOI:** 10.1371/journal.pone.0223579

**Published:** 2019-10-09

**Authors:** Umedjon Ibragimov, Stephanie Beane, Samuel R. Friedman, Kelli Komro, Adaora A. Adimora, Jessie K. Edwards, Leslie D. Williams, Barbara Tempalski, Melvin D. Livingston, Ronald D. Stall, Gina M. Wingood, Hannah L. F. Cooper

**Affiliations:** 1 Department of Behavioral Sciences and Health Education, Rollins School of Public Health, Emory University, Atlanta, GA, United States of America; 2 National Development and Research Institutes Inc, New York, NY, United States of America; 3 Department of Epidemiology, Gillings School of Global Public Health, University of North Carolina at Chapel Hill, Chapel Hill, NC, United States of America; 4 Division of Infectious Diseases, School of Medicine, University of North Carolina at Chapel Hill, Chapel Hill, NC, United States of America; 5 Division of Community Health Sciences, University of Illinois at Chicago School of Public Health, Chicago, IL, United States of America; 6 Department of Behavioral and Community Health Sciences and Department of Infectious Diseases and Microbiology, Graduate School of Public Health, University of Pittsburgh, Pittsburgh, PA, United States of America; 7 Department of Sociomedical Sciences, Columbia University, New York, NY, United States of America; Robert Koch Institut, GERMANY

## Abstract

Prior research has found that places and people that are more economically disadvantaged have higher rates and risks, respectively, of sexually transmitted infections (STIs). Economic disadvantages at the level of places and people, however, are themselves influenced by economic policies. To enhance the policy relevance of STI research, we explore, for the first time, the relationship between state-level minimum wage policies and STI rates among women in a cohort of 66 large metropolitan statistical areas (MSAs) in the US spanning 2003–2015. Our annual state-level minimum wage measure was adjusted for inflation and cost of living. STI outcomes (rates of primary and secondary syphilis, gonorrhea and chlamydia per 100,000 women) were obtained from the CDC. We used multivariable hierarchical linear models to test the hypothesis that higher minimum wages would be associated with lower STI rates. We preliminarily explored possible socioeconomic mediators of the minimum wage/STI relationship (e.g., MSA-level rates of poverty, employment, and incarceration). We found that a $1 increase in the price-adjusted minimum wage over time was associated with a 19.7% decrease in syphilis rates among women and with an 8.5% drop in gonorrhea rates among women. The association between minimum wage and chlamydia rates did not meet our cutpoint for substantive significance. Preliminary mediation analyses suggest that MSA-level employment among women may mediate the relationship between minimum wage and gonorrhea. Consistent with an emerging body of research on minimum wage and health, our findings suggest that increasing the minimum wage may have a protective effect on STI rates among women. If other studies support this finding, public health strategies to reduce STIs among women should include advocating for a higher minimum wage.

## Introduction

Women in the US continue to bear high burden of sexually transmitted infections (STIs), and recent surveillance data show steady increases in rates of reported cases of primary and secondary syphilis, chlamydia, and gonorrhea among women (by 155.6%, 11.1% and 39.4% respectively over 2013–2017) [1]. Women are particularly vulnerable to STIs because of their physiological susceptibility and asymptomatic course of some common STIs [2]. Significant complications of STIs among women, including pelvic inflammatory disease, can lead to a higher risk of infertility and ectopic pregnancy [3]. The drastic increase in the number of congenital syphilis cases (by 253.6% in 2013–2017) also highlights the urgent need to prevent STIs among women of reproductive age in the US [4].

In recent years, research on the determinants of STIs has shifted its focus from individual-level characteristics to upstream structural factors that create population-level vulnerability and resilience to STIs [5, 6]. This line of inquiry has found that several structural factors, including poverty rates and income inequality in geographic areas, help propagate STIs [5, 7–10]. Poverty, low-wage jobs, income inequality, and other economic structural factors may spread STIs by creating high-risk partner pools, facilitating transactional sex, and perhaps also undermining women’s sexual agency [5, 11–16]. Empirical evidence suggests that area-level poverty may have population-wide effect by increasing the risk of STI transmission for individuals irrespective of their individual sexual practices or socioeconomic status [17].

These place-based socioeconomic factors, however, are themselves shaped by economic policies, as well as other distal structural processes (e.g., demographic, social, and political factors) [18–20]. To enhance public health responses to STIs, Leichliter et al. have called for identifying the policies that affect vulnerability to STIs by shaping poverty, economic inequality, wages, and other economic structural factors [6]. Studying policies as primary exposures (while recognizing that the relationships between policy reforms and socioeconomic conditions are complex and multi-directional) enhances public health by directly informing legislative debates; literature on the structural determinants of health, and of STIs in particular, increasingly promotes a policy focus [6, 21, 22].

Minimum wage laws may powerfully shape STI rates in the US. Proponents of a higher minimum wage argue that increasing wages will help reduce poverty and decrease economic inequality (particularly for women) [23, 24]. These structural changes may reduce STI rates.[5] Studies in US and other industrialized countries suggest that higher minimum wages are associated with a range of better health outcomes across the life course, including lower rates of adolescent pregnancy, infant mortality, low birthweight, and chronic disease [25–27]. Our exploratory ecological analysis answers Leichlitter et al.’s [6] call to enhance the policy relevance of research on the structural determinants of STIs by testing the hypothesis that higher state minimum wage levels will be associated with lower STI rates among women living in large US metropolitan statistical areas (MSAs) between 2003 and 2015. The study is guided by Buot’s model of socioeconomic determinants of HIV risk [28], which posits that poverty, educational attainment, income, and social inequality influence risky sexual practices and sexual network patterns by shaping community and family instability, crime, and incarceration.

## Methods

This is a cohort study examining the relationship between local cost of living adjusted state minimum wage and annual (2003–2015) STI rates in large US MSAs. An MSA is defined as an area containing “…at least one urbanized area that has a population of at least 50,000 …” and “…counties having a high degree of social and economic integration with the central [urbanized] county…” [29 p. 82238]. To meet the eligibility criterion for this study, an MSA had to have a population size > 500,000 in 1993, the beginning of the parent study. Ninety-six MSAs met this eligibility criterion, but data on the cost of living (needed to construct the minimum wage variable) and EITC were unavailable for 30 MSAs, reducing our final cohort to 66 MSAs. The parent study spans 1992–2015 [30], so we used 1990 MSA boundaries.

### Measures

#### Outcome

For each MSA, and year (2003–2015), the CDC provided data on rates of new diagnoses of (1) primary and secondary syphilis, (2) gonorrhea, and (3) chlamydia among women per 100,000 female residents. We used these data to create MSA- and year-specific measures for each STI.

#### Independent variables

We used Komro et al.’s minimum wage database to calculate the annual state minimum wage. As described by Komro et al. [25], to measure the state minimum wage over time legal coders reviewed legislation and created a database of monthly state minimum wage values. We averaged these monthly values to create the annual minimum wage for each state (hereafter referred to as the “minimum wage”) for 2000–2014; we reached back to 2000 to allow a temporal lag between independent variables and the outcomes. Minimum wage was adjusted for inflation using the consumer price index, and for local cost of living using an index created by the Council for Community and Economic Research. Fifteen MSAs in our cohort crossed state boundaries. In these cases, we used minimum wage data for the state where most MSA residents lived.

#### Confounders

Drawing on past literature, we identified sociodemographic characteristics of MSAs that might confound the minimum wage/STI rate relationship. These characteristics included population density; racial/ethnic composition; age composition; residential racial segregation; adult sex ratios; percentage of female-headed households, and teen birth rates [10, 31–36]. We also included the state-level percent of supplemental nutrition assistance program (SNAP) recipients, percent of temporary assistance for needy families (TANF) recipients, and the earned income tax credit (EITC) rate and EITC refund policy as potential confounders since SNAP, TANF and EITC may influence both total income of low-income populations and their health status [37, 38]. Potential confounders capturing access to services were: access to healthcare services (insurance rates and health provider shortage areas); government expenditures on health, community and housing; and an estimate of syringe exchange coverage (assessed as the quantity of syringes exchanged via syringe service programs per 10,000 persons who inject drugs) [5, 39–42]. We included syringe coverage to account for the possibility of parenteral transmission of syphilis among people who inject drugs[43], and because these programs distribute large numbers of condoms [39].

#### Possible mediators

To support future hypothesis testing about pathways linking minimum wage to STIs, we conducted preliminary mediation analyses. We selected the following MSA-level socioeconomic characteristics based on Buot’s model [28], economics literature on the impacts of the minimum wage on socioeconomic characteristics, and public health literature on socioeconomic predictors of STIs: poverty rates for all individuals and for female-headed households, [7, 23] income inequality (the Gini index),[8, 9, 24], [44] total and female employment rate,[5, 10, 45, 46] educational attainment (high school diploma) for all adults and for women,[5, 10, 47] male and female incarceration rate,[48, 49] and affordable housing (percent of low income households with rent more than 30% of income) [12, 13, 50].

S1 Table contains additional information on measures and data sources.

### Analysis

We used descriptive statistics to summarize each variable’s distribution. We used a 3-stage model-building process for each STI outcome, first modeling temporal changes in the STI (Stage 1), and then conducting multilevel bivariate (Stage 2) and multivariable analyses (Stage 3). We chose hierarchical linear modelling (HLM) as our analytical approach since it allowed us to model the 13-year trajectory of STI rates within MSAs over time, assess variability in these trajectories across MSAs, and account for correlation of clustered observations (e.g., time within MSAs).[51]

Our study sought to analyze a census of all MSAs with a population of 500,000 or larger; therefore, assumptions regarding interpretation of *p*-values and confidence intervals do not hold [52, 53]. We therefore determined *substantive* significance using the magnitude of association. Associations were deemed substantively significant if a standardized coefficient for a variable was ≥|0.10| in final models [54].

#### Stage 1: Modeling change in each outcome over time

We modeled annual (2003–2015) rates for each STI separately. We used a log transformation to linearize the relationship between minimum wage and syphilis rates because of skewed distribution of this outcome. Gonorrhea and chlamydia rates were normally distributed and did not require transformation. All models (Stages 1–3) used 3-level HLM to account for the clustering of annual observations within MSAs, and of MSAs within states (28% of states had only one MSA; however, according to HLM literature, this proportion of sparse observations is not expected to introduce bias into the fixed effects) [51, 55]. To quantify the variability of each outcome within and between MSAs, we assessed covariance parameters and calculated intraclass correlation coefficients (ICCs) [56]. For all STIs, nested models yielded the smallest Akaike information criterion (AIC) when time since baseline was modeled with fixed cubic and random quadratic time.

#### Stage 2: Bivariate models

We centered each time-varying covariate at baseline to aid interpretation [51]. Centering produced variable “dyads” for each time-varying covariate, with one variable capturing the baseline value and another capturing annual change in the variable since baseline, hereinafter referred to as the annual change since baseline. We report findings using magnitudes of association and standardized coefficients, obtained by creating z scores for variables, in bivariate and multivariate analysis and report confidence intervals as a heuristic guide [52, 53].

We did not expect minimum wage changes to have an instantaneous effect on STIs, and so we tested bivariate models with a 1-, 2-, and 3-year lag between minimum wage and each STI outcome. The 1-year lag had the largest standardized coefficient, so we proceeded with a 1-year lag for all bivariate and multivariate models.

We built models for each STI separately. First, we used HLM models to regress annual STI rates on time (operationalized as years since baseline) and the lagged minimum wage dyad (baseline and annual change since baseline). Next, we added possible confounders, one “dyad” at a time. To determine which putative covariate might actually confound the minimum wage/STI relationship, we used a rule of thumb—specifically, that the magnitude of the minimum wage/STI relationship changed by ≥10% across the models with and without the putative confounder dyad. This is an appropriate strategy given the relatively small sample size and expected small effect size [57]. Note that because the minimum wage was operationalized using a baseline and a change since baseline dyad, we determined whether 10% change occurred in the *absolute magnitude of the sum of the standardized coefficients for the minimum wage dyad*.

#### Stage 3: Multivariable analysis

We used multivariable HLM models to test the relationship between the minimum wage and annual STI rates, controlling for demographic and access to service confounders selected from the bivariate analyses. Since the variance component for state became 0 in the multivariate models and, therefore, unnecessary, we present the multivariate models without state random effects [56]. We tested the models for multicollinearity using condition index and variance decomposition proportions [58, 59]. To aid interpretation, we re-ran the final multivariate models with unstandardized minimum wage and STI variables to assess changes in STI rates per $1 change in minimum wage. Since we log transformed the syphilis outcome to normalize and linearize its relationship with covariates, we used back transformation [60] to report the percent change in syphilis rates. To calculate the percent change for gonorrhea and chlamydia per $1 change in the minimum wage, we divided the unstandardized coefficients by median 2015 STI rates and multiplied by 100. Sensitivity analysis involved testing the multivariate models without influential observations selected based on Cook’s Distance criterion [61, 62].

Once we had constructed the final multivariable model, we conducted a preliminary exploration of covariates that might mediate the minimum wage/STI relationship to inform future research. We tested separate models for each covariate that met the criteria for inclusion in bivariate analysis by adding each of them to the final multivariable model; to meet this bivariate criterion, the potential mediator had to generate a ≥10% change in the absolute magnitude of the standardized coefficient for minimum wage in the bivariate model. If the absolute value of the standardized coefficient for either or both of the substantively significant minimum wage variable (i.e., baseline or change since baseline or both) changed by ≥10% after adding a potential mediator, we interpreted it as a preliminary indication of mediation [63].

We conducted our analyses in SAS 9.4 (SAS Institute Inc., Cary, NC).

## Results

The adjusted median minimum wage was $6.90 in 2002 (25^th^ and 75^th^ percentiles: $6.54, $7.23) and this median value increased by 50 cents between 2002 and 2014 (25th and 75th percentiles: $0.47, $1.11). In 2003, at baseline, the median rate of newly diagnosed primary and secondary syphilis was 0.5/100,000 women (25^th^ and 75^th^ percentiles: 0.3/100,000, 1.5/100,000); the median change in the rate of newly diagnosed syphilis cases from 2003 to 2015 was 0.3 per 100,000 women (25^th^ and 75^th^ percentiles: -0.3/100,000, 1.3/100,000). The median rate of newly diagnosed cases of gonorrhea among women was 142.9/100,000 in 2003 (25^th^ and 75^th^ percentiles: 96.3/100,000, 197.3/100,000), and median change from 2003 to 2015 was -11.0/100,000 women (25^th^ and 75^th^ percentiles: -49.2/100,000, 12.1/100,000). In 2003 the median chlamydia rate was 512.0/100,000 women (25^th^ and 75^th^ percentiles: 395.3/100,000, 587.4/100,000); the median change in the chlamydia rate from 2003 to 2015 was 179.6/100,000 women (25^th^ and 75^th^ percentiles: 123.4/100,000, 231.2/100,000) (Table 1). Descriptive statistics for potential confounders are presented in Table 1; descriptive statistics for potential mediators are presented in S2 Table.

**Table 1:** Descriptive statistics for rates of primary and secondary syphilis, gonorrhea, and chlamydia among females per 100,000 over time and possible structural correlates: 66 large US metropolitan statistical areas, 2003–2015^a^.

Variables	Mean	Std Dev	Median	25th Pctl	75th Pctl
Primary and secondary syphilis rates per 100,000 females					
*Baseline (2003)*	1.03	1.20	0.52	0.25	1.46
*Change between 2003 and 2015*	0.64	2.63	0.25	-0.34	1.30
Gonorrhea rates per 100,000 females					
*Baseline (2003)*	145.84	65.73	142.91	96.25	197.28
*Change between 2003 and 2015*	-18.44	43.99	-10.95	-49.15	12.06
Chlamydia rates per 100,000 females					
*Baseline (2003)*	505.91	139.05	512.00	395.29	587.44
*Change between 2003 and 2015*	180.82	98.04	179.62	123.36	231.22
**Correlates**					
Cost of living and inflation adjusted minimum wage					
*Lagged baseline (2002)*	6.87	0.78	6.90	6.54	7.23
*Change between 2002 and 2014*	0.60	0.51	0.50	0.47	1.11
% living in a health provider shortage area					
*Lagged baseline (2002)*	14.33	15.42	9.95	3.84	21.30
Syringes exchanged per injection drug users					
*Lagged baseline (2002)*	1,732.05	6,000.60	0.00	0.00	849.88
Population density per square mile					
*Lagged baseline (2002)*	667.65	1006.96	470.24	316.82	699.82
*Change between 2002 and 2014*	73.01	65.65	60.58	30.15	106.62
% residents 15–29					
*Lagged baseline (2002)*	30.97	2.47	30.76	29.67	32.88
*Change between 2002 and 2014*	0.00	0.01	0.00	-0.01	0.01
% Hispanic residents					
*Lagged baseline (2002)*	13.61	15.99	6.56	3.25	19.11
*Change between 2002 and 2014*	3.67	1.90	3.22	2.33	4.68
% non-Hispanic black residents					
*Lagged baseline (2002)*	14.17	9.05	12.19	7.37	18.85
*Change between 2002 and 2014*	0.00	0.01	0.00	-0.00	0.01
% female-headed households					
*Lagged baseline (2002)*	13.24	1.96	13.08	12.14	14.17
*Change between 2002 and 2014*	0.19	0.84	0.22	-0.28	0.80
Black isolation index					
*Lagged baseline (2002)*	40.70	17.90	41.64	30.84	53.61
*Change between 2002 and 2014*	-4.81	2.99	-4.76	-6.95	-2.73
Hispanic sex ratio					
*Lagged baseline (2002)*	1.19	0.22	1.15	1.03	1.30
*Change between 2002 and 2014*	-0.12	0.20	-0.07	-0.19	-0.00
Non-Hispanic black sex ratio					
*Lagged baseline (2002)*	0.91	0.14	0.86	0.83	0.96
*Change between 2002 and 2014*	0.02	0.04	0.02	-0.00	0.04
Teen birth rate					
*Lagged baseline (2002)*	21.96	5.64	21.86	18.12	25.09
*Change between 2002 and 2014*	-10.15	3.20	-10.34	-12.26	-7.96
% uninsured residents					
*Lagged baseline (2002)*	16.47	5.58	15.66	11.94	19.86
*Change between 2002 and 2014*	-3.09	3.26	-2.55	-5.40	-0.59
% uninsured women					
*Lagged baseline (2002)*	15.13	5.44	14.10	11.17	18.68
*Change between 2002 and 2014*	-2.96	3.38	-2.33	-4.81	-0.56
Cost of living and inflation adjusted health expenditures per capita					
*Lagged baseline (2002)* *Lagged endline (2014)*	114.73	84.40	77.80	47.94	178.85
*Change between 2002 and 2014*	-8.29	96.77	2.80	-20.99	25.79
Cost of living and inflation adjusted community and housing development expenditures per capita					
*Lagged baseline (2002)*	120.56	45.38	110.39	91.11	150.52
*Change between 2002 and 2014*	-60.81	651.05	16.98	-12.72	36.97
State earned income tax credit (EITC) as a percentage of federal EITC					
*Lagged baseline (2002)*	3.25	7.46	0.00	0.00	0.00
*Change between 2002 and 2014*	3.43	6.29	0.00	0.00	5.00
State EITC refund policy					
*% (n) of MSAs in states with EITC refund in 2002*	18.2% (12)				
*% (n) of MSAs in states with EITC refund in 2014*	30.3% (20)				
State percent of SNAP recipients					
*Lagged baseline (2002)*	6.94	2.09	6.56	5.17	7.59
*Change between 2002 and 2014*	8.15	1.86	8.50	6.61	9.00
*S*tate percent of TANF recipients					
*Lagged baseline (2002)*	1.75	0.94	1.67	1.16	2.09
*Change between 2002 and 2014*	-0.58	0.74	-0.42	-0.94	-0.21

^a^ 2003 to 2015 is the timeframe for the STI outcome. Correlates were lagged 1 year and reflect 2002–2014. Descriptive statistics were assessed for 68 MSAs with available data on price-adjusted minimum wage (for EITC variables data were available for 66 MSAs only).

Note: Correlates were lagged one year because we did not expect a change in the correlates to have an instantaneous effect on the outcome.

Analysis of the ICC for each STI suggested that a meaningful proportion of the outcome variance was attributable to differences *between* MSAs (syphilis = 0.42, gonorrhea = 0.78, chlamydia = 0.67) and *between* states (syphilis = 0.33, gonorrhea = 0.57, chlamydia = 0.30), thus justifying multilevel analysis.

### Syphilis

Bivariate analyses found that baseline minimum wage was positively correlated with log syphilis rate (standardized coefficient [B] = 0.07) and the minimum wage annual change since baseline was inversely related to the (logged) syphilis rate (B = -0.09; Table 2).

**Table 2:** Results of hierarchical linear bivariate regressions of rates of primary and secondary syphilis, gonorrhea, and chlamydia among women per 100,000 over time and possible structural correlates: 66 large US metropolitan statistical areas, 2003–2015^a^.

Correlates	Log syphilis bivariate models^b^ standardized coefficient (95% CI)	Gonorrhea bivariate models^b^ standardized coefficient (95% CI)	Chlamydia bivariate models^b^ standardized coefficient (95% CI)
Cost of living and inflation adjusted minimum wage			
*Lagged baseline (2002)*	0.07 (-0.12, 0.26)	0.05 (-0.18, 0.29)	0.06 (-0.17, 0.29)
*Annual change since 2002*	-0.09 (-0.18, 0)	-0.10 (-0.15, -0.05)	0.04 (-0.01, 0.08)
% living in a health provider shortage area			
*Lagged baseline (2002)*	0.01 (-0.17, 0.18)	0.15 (-0.06, 0.37)	0.30 (0.09, 0.51)
Syringes exchanged per injection drug users			
*Lagged baseline (2002)*	-0.01 (-0.21, 0.19)	0.02 (-0.22, 0.26)	0.06 (-0.17, 0.28)
Population density per square mile			
*Lagged baseline (2002)*	-0.09 (-0.28, 0.10)*	-0.08 (-0.31, 0.14)	0.05 (-0.18, 0.28)*
*Annual change since 2002*	0.15 (-0.02, 0.33)*	-0.01 (-0.14, 0.12)	0.03 (-0.09, 0.15)*
% residents aged 15–29			
*Baseline (2002)*	0.12 (-0.09, 0.33)	0.16 (-0.10, 0.41)*	0.47 (0.27, 0.68)
*Annual change since 2002*	0.02 (-0.11, 0.14)	-0.01 (-0.10, 0.08)*	0.13 (0.05, 0.20)
% non-Hispanic black residents			
*Lagged baseline (2002)*	0.36 (0.19, 0.54)*	0.63 (0.44, 0.81)*	0.51 (0.31, 0.70)*
*Annual change since 2002*	-0.04 (-0.14, 0.06)*	0.01 (-0.06, 0.08)*	-0.01 (-0.07, 0.06)*
% Hispanic residents			
*Lagged baseline (2002)*	-0.09 (-0.31, 0.13)*	-0.38 (-0.63, -0.13)*	0.02 (-0.23, 0.26)
*Annual change since 2002*	0.08 (-0.12, 0.29)*	0.00 (-0.15, 0.15)*	0.04 (-0.10, 0.17)
% of female-headed households			
*Lagged baseline (2002)*	0.29 (0.12, 0.45)*	0.47 (0.29, 0.66)*	0.70 (0.54, 0.85)*
*Annual change since 2002*	0.04 (-0.08, 0.17)*	0.12 (0.03, 0.21)*	0.08 (0.00, 0.16)*
Black isolation index			
*Lagged baseline (2002)*	0.31 (0.11, 0.51)*	0.62 (0.42, 0.83)*	0.28 (0.05, 0.51)*
*Annual change since 2002*	0.00 (-0.17, 0.17)*	0.08 (-0.05, 0.20)*	0.08 (-0.03, 0.20)*
Hispanic sex ratio			
*Lagged baseline (2002)*	0.28 (0.05, 0.5)*	0.53 (0.3, 0.76)*	0.25 (0.02, 0.48)*
*Annual change since 2002*	0.1 (-0.05, 0.25)*	0.07(-0.04, 0.18)*	0.04 (-0.06, 0.15)*
Non-Hispanic black sex ratio			
*Lagged baseline (2002)*	-0.19 (-0.37, -0.01)*	-0.45 (-0.65, -0.24)*	-0.10 (-0.33, 0.13)*
*Annual change since 2002*	-0.06 (-0.19, 0.07)*	0.06 (-0.03, 0.16)*	0.05 (-0.04, 0.13)*
Births per 1,000 females aged 10–19			
*Lagged baseline (2002)*	0.37 (0.17, 0.57)*	0.42 (0.17, 0.66)	0.49 (0.27, 0.71)*
*Annual change since 2002*	0.16 (0, 0.31)*	0.11 (0.02, 0.20)	0.19 (0.11, 0.27)*
% uninsured residents			
*Lagged baseline (2002)*	0.16 (-0.1, 0.42)	-0.27 (-0.57, 0.02)	-0.11 (-0.36, 0.14)
*Annual change since 2002*	-0.03 (-0.14, 0.07)	0.05 (-0.02, 0.12)	-0.05 (-0.11, 0.01)
% uninsured women			
*Lagged baseline (2002)*	0.19 (-0.06, 0.44)	-0.25 (-0.54, 0.04)*	-0.13 (-0.38, 0.12)
*Annual change since 2002*	0.00 (-0.11, 0.10)	0.06 (-0.01, 0.13)*	-0.03 (-0.09, 0.03)
Cost of living and inflation adjusted health expenditures per capita			
*Lagged baseline (2002)*	-0.19 (-0.44, 0.06)	-0.01 (-0.31, 0.29)	0.02 (-0.20, 0.24)
*Annual change since 2002*	0.04 (-0.04, 0.12)	-0.03 (-0.08, 0.03)	-0.03 (-0.08, 0.02)
Cost of living and inflation adjusted community and housing development expenditures per capita			
*Lagged baseline (2002)*	0.01 (-0.18, 0.21)	0.07 (-0.17, 0.30)	0.14 (-0.08, 0.36)*
*Annual change since 2002*	0.04 (-0.03, 0.12)	-0.01 (-0.07, 0.04)	-0.03 (-0.08, 0.03)*
State earned income tax credit (EITC) as a percentage of federal EITC			
*Lagged baseline (2002)*	-0.18 (-0.41, 0.06)*	-0.19 (-0.47, 0.10)*	-0.05 (-0.29, 0.18)*
*Annual change between 2002 and 2014*	-0.01 (-0.11, 0.08)*	-0.04 (-0.10, 0.02)*	0.01 (-0.05, 0.06)*
State EITC refund policy			
*Refundable EITC in 2002 (unstandardized coefficient*.*)*	-0.39 (-0.69, -0.09)*	-0.27 (-0.47, -0.06)*	-0.12 (-0.30, 0.07)*
*Interaction of refund policy and time*	-0.02 (-0.09, 0.12)*	0.02 (-0.12, 0.16)*	-0.07 (-0.22, 0.07)*
State percent of SNAP recipients			
*Lagged baseline (2002)*	0.35 (0.11, 0.59)*	0.40 (0.12, 0.69)*	0.20 (-0.07, 0.46)* 8
*Annual change since 2002*	0.0 (-0.11, 0.11)*	0.04 (-0.03, 0.10)*	-0.05 (-0.11, 0.01)*
*S*tate percent of TANF recipients			
*Lagged baseline (2002)*	-0.16 (-0.42, 0.09)	-0.19 (-0.49, 0.1)	0.03 (-0.21, 0.28)
*Annual change since 2002*	-0.02 (-0.13, 0.09)	-0.01 (-0.08, 0.06)	0.01 (-0.05, 0.07)

^a^ 2003 to 2015 is the timeframe for the STI outcome. Correlates were lagged 1 year and reflect 2002–2014. Bivariate models were assessed for 68 MSAs except for the models with EITC variables, where data were available for 66 MSAs only.

Note: Correlates were lagged one year because we did not expect a change in the correlates to have an instantaneous effect on the outcome.

^b^ The bivariate models include time, state as a random effect, and standardized covariate dyad (baseline and change values).

*Covariates that passed a priori threshold at bivariate stage (changing the standardized coefficients for minimum wage and relevant STIs by ≥ 10%) and were included to the multivariable models.

In multivariable models that controlled for multiple confounders, the magnitude of the relationship between baseline minimum wage and log syphilis decreased and changed direction (B = -0.09) and is just below our cut-point of >|0.10| for substantive significance (Table 3). The relationship between the minimum wage annual change since baseline and syphilis rates remained the same (B = -0.09) across bivariate and multivariate models and is also just below our standard for substantive significance >|.10|. Back transformation of the unstandardized coefficient of log-transformed syphilis outcome at baseline (b = -0.21) and for the annual change since baseline (b = -0.22) shows that a $1 increase in the minimum wage annual change since baseline is associated with a 18.6% (baseline) and 19.7% (annual change since baseline) decrease in the syphilis rate among women (or 0.30 and 0.27 cases fewer per 100,000 women based on 2015 median rate).

**Table 3:** Results of hierarchical linear multivariate regressions of rates of primary and secondary syphilis, gonorrhea, and chlamydia among women per 100,000 over time and possible structural correlates: 66 large US metropolitan statistical areas, 2003–2015^a^.

Correlates	Log syphilis mulitvariate models^b^ standardized coefficient (95% CI)	Gonorrhea multivariate models^b^ standardized coefficient (95% CI)	Chlamydia multivariate models^b^ standardized coefficient (95% CI)
Cost of living and inflation adjusted minimum wage			
*Lagged baseline (2002)*	-0.09 (-0.34, 0.16)	0.03 (-0.13, 0.18)	0.01 (-0.22, 0.23)
*Annual change since 2002*	-0.09 (-0.17, 0.0)	-0.10 (-0.15, -0.05)	0.03 (-0.01, 0.08)
Time			
*Years since 2002*	-0.14 (-0.45, 0.16)	-0.11 (-0.29, 0.08)	0.54 (0.38, 0.71)
*Fixed cubic time*	0.12 (0.01, 0.23)	0.02 (-0.04, 0.08)	-0.07 (-0.14, 0.0)
*Random quadratic time*	0.11 (0.04, 0.18)	0.02 (-0.02, 0.07)	-0.04 (-0.08, -0.01)
Population density per square mile			
*Lagged baseline (2002)*	-0.16 (-0.43, 0.11)	--	-0.07 (-0.31, 0.17)
*Annual change since 2002*	0.21 (0.04, 0.38)	--	0.06 (-0.05, 0.17)
% residents aged 15–29			
*Baseline (2002)*	--	0.25 (0.06, 0.43)	0.21 (0.03, 0.40)
*Annual change since 2002*	--	-0.02 (-0.1, 0.07)	0.05 (-0.04, 0.13)
% Hispanic residents			
*Lagged baseline (2002)*	-0.11 (-0.36, 0.15)	-0.33 (-0.59, -0.06)	--
*Annual change since 2002*	0.07 (-0.12, 0.26)	0.01 (-0.12, 0.14)	--
% of female-headed households			
*Lagged baseline (2002)*	0.16 (-0.07, 0.38)	0.28 (0.09, 0.47)	0.57 (0.36, 0.78)
*Annual change since 2002*	0.08 (-0.05, 0.21)	0.16 (0.08, 0.25)	0.06 (-0.03, 0.15)
Black isolation index			
*Lagged baseline (2002)*	0.13 (-0.18, 0.44)	0.46 (0.19, 0.73)	0.17 (-0.12, 0.46)
*Annual change since 2002*	-0.07 (-0.24, 0.1)	0.06 (-0.06, 0.18)	0.04 (-0.08, 0.16)
Hispanic sex ratio			
*Lagged baseline (2002)*	-0.02 (-0.25, 0.2)	0.24 (0.06, 0.42)	0.28 (0.11, 0.45)
*Annual change since 2002*	0.10 (-0.06, 0.26)	0.06 (-0.04, 0.16)	0.02 (-0.09, 0.13)
Non-Hispanic black sex ratio			
*Lagged baseline (2002)*	-0.05 (-0.3, 0.19)	-0.02 (-0.23, 0.2)	0.03 (-0.18, 0.24)
*Annual change since 2002*	-0.04 (-0.18, 0.1)	0.08 (-0.02, 0.18)	0.04 (-0.06, 0.14)
Births per 1,000 females aged 10–19			
*Lagged baseline (2002)*	0.35 (0.12, 0.59)	--	0.18 (-0.02, 0.37)
*Annual change since 2002*	0.17 (0.02, 0.33)	--	0.14 (0.05, 0.23)
% uninsured residents			
*Lagged baseline (2002)*	--	-0.04 (-0.25, 0.16)	--
*Annual change since 2002*	--	0.02 (-0.05, 0.08)	--
Cost of living and inflation adjusted community and housing development expenditures per capita			
*Lagged baseline (2002)*	--	--	-0.02 (-0.18, 0.14)
*Annual change since 2002*	--	--	-0.05 (-0.1, -0.01)
State earned income tax credit (EITC) as a percentage of federal EITC			
*Lagged baseline (2002)*	0.04 (-0.16, 0.24)	-0.14 (-0.3, 0.02)	0.06 (-0.10, 0.22)
*Annual change between 2002 and 2014*	0.03 (-0.06, 0.12)	-0.03 (-0.09, 0.03)	0.03 (-0.03, 0.08)
State EITC refund policy			
*Refundable EITC in 2002 (unstandardized coefficient)*	-0.31 (-0.64, 0.02)	-0.14 (-0.36, 0.07)	-0.12 (-0.31, 0.07)
*Interaction of refund policy and time*	-0.05 (-0.24, 0.14)	0.01 (-0.12, 0.13)	-0.06 (-0.18, 0.06)
State percent of SNAP recipients			
*Lagged baseline (2002)*	0.20 (-0.02, 0.42)	0.03 (-0.17, 0.22)	-0.20 (-0.4, 0.0)
*Annual change since 2002*	0.04 (-0.06, 0.14)	0.04 (-0.02, 0.10)	-0.03 (-0.08, 0.03)

^a^ 2003 to 2015 is the timeframe for the STI outcome. Correlates were lagged 1 year and reflect 2002–2014.

Note: Correlates were lagged one year because we did not expect a change in the correlates to have an instantaneous effect on the outcome.

^b^ Multivariate models include correlates that changed the focal exposure (baseline minimum wage and change since 2002) by 10% or more.

As noted, we preliminarily explored potential mediation of this relationship. None of the covariates explored here met our a priori threshold for possible mediation (i.e., the magnitude of the minimum wage/STI relationship changed by ≤10% when each putative mediator was added both to the bivariate and multivariate models; see S3 and S4 Tables).

### Gonorrhea

In bivariate models, baseline minimum wage was positively correlated with gonorrhea rates (B = 0.05) and an inverse relationship existed between the minimum wage annual change since baseline and gonorrhea (B = -0.10; Table 2). In multivariable models controlling for several confounders, the magnitude of association between the baseline minimum wage and gonorrhea rates became very small (B = 0.03; Table 3). The magnitude of association between change in minimum wage and gonorrhea rates remained -0.10, meeting our cutpoint for substantive significance. Based on unstandardized coefficients, a $1 increase in the minimum wage annual change since baseline (b = -11.13) was associated with an 8.5% drop in the median 2015 gonorrhea rate (or 11 cases fewer per 100,000 women based on 2015 median rate).

Preliminary tests of potential mediators show that the percent of employed females met our exploratory criterion for mediation (S3 and S4 Tables). Adding this covariate reduced the absolute value of the standardized coefficient for the minimum wage annual change since baseline by 18.69% (B for minimum wage change = -0.08). Post hoc analyses showed that increasing the minimum wage was associated with a modest increase in employment rates among women (B = 0.05) in the bivariate model, and that increase in employment rate among women over time was associated with reduced gonorrhea rate (B for employment annual change since baseline = -0.10) in the multivariate model.

### Chlamydia

Bivariate analyses revealed that baseline minimum wage and the minimum wage annual change since baseline were both positively correlated with chlamydia rates (B = 0.06 and B = 0.04, respectively; Table 2). When the model controlled for multiple possible confounders, the magnitudes of the relationship between chlamydia and the minimum wage baseline (B = 0.01) and annual change since baseline (B = 0.03) slightly decreased remaining below our cut point for substantive significance (Table 3).

Due to large racial/ethnic disparities in STI distributions, and the possibility that the racial/ethnic composition of MSAs and minimum wage policies may have common unmeasured antecedents (e.g. structural racism), we also re-ran the models with the percent of non-Hispanic black residents as a covariate. The percent of residents who were non-Hispanic black was highly correlated with residential segregation, so we did not test both variables in the same models to avoid multicollinearity. Replacing black isolation with the percent of the population who are non-Hispanic black in the models did not substantially change the results–the standardized coefficients for the minimum wage baseline and annual change since baseline were -0.07 and -0.09 for syphilis, -0.03 and -0.10 for gonorrhea, and -0.003 and 0.03 for chlamydia correspondingly. Excluding influential observations (n = 5 for syphilis, n = 3 gonorrhea, n = 6 for chlamydia) did not substantially change the coefficients for the association between the minimum wage annual change since baseline and STIs (the greatest absolute change in these coefficients was 0.03). However, the coefficients for baseline minimum wage did change for syphilis (B = -0.03) suggesting that some of our findings may be influenced by MSA outliers.

## Discussion

This analysis extends the emerging line of research on possible health effects of the minimum wage. Consistent with other studies in this emerging area that have found that higher minimum wages may be protective [25–27], we found that MSAs in states with higher minimum wages have lower rates of two common reportable STIs among women. Specifically, we found that a $1 increase in the adjusted minimum wage over time was associated with 12.2% drop in the syphilis rate among women (0.16 fewer cases per 100,000 based on 2015 median rates) and with a 7.4% drop in gonorrhea rates (or 9.7 fewer cases/100,000). When considered at a population level, if all states in our sample enacted this increase, the impact would be substantial, if they are causal: about 50% of all women in the US lived in one of the 66 MSAs in our cohort. We found no substantively significant association between minimum wage (either baseline or change over time) and chlamydia rates.

One key next step in this line of research is to understand the pathways through which minimum wage may influence STIs. Our preliminary exploration of possible mediation suggests that increasing the minimum wage might reduce gonorrhea rates among women by improving MSA-level employment rates among women. While most economic studies found negative or zero effect of minimum wage on employment rates [64, 65], a small number of studies suggest that higher minimum wage may increase employment in certain industries or states [45, 46]. Consequently, when more women are employed (and minimum wages are higher), women may have greater economic security to support engaging in protective practices like negotiating condom use, avoiding transactional sex, and seeking medical care for STI testing and treatment [66, 67]. In MSAs with higher employment rates among women, these practices may become more normative, thus helping to reduce STI risks at population level. Still, this potential pathway should be considered with caution because of scant empirical evidence on positive effects of higher minimum wages on employment.

Variations in the magnitudes of relationships between minimum wage and STI rates across STIs might be explained by racial/ethnic differences in (1) employment patterns, and (2) the racial/ethnic composition of the population of women diagnosed with gonorrhea, syphilis, and chlamydia. Specifically, white women are less likely to work in jobs covered by minimum wage laws than black women [68]. White women also constitute a higher proportion of reported chlamydia cases than they do of gonorrhea and syphilis diagnoses [69]. These racialized patterns of minimum wage coverage and STI composition might explain why we found relationships between minimum wage levels and gonorrhea and syphilis (black women constitute a higher percentage of women diagnosed with these STIs, and also are more likely to work in jobs covered by minimum wage laws), but not chlamydia (white women constitute a higher percentage of women diagnosed with chlamydia, and are less likely to work in jobs covered by minimum wage laws). Future research should explore this interpretation. Alternatively, chlamydia is more evenly distributed across the US,[70] while syphilis and gonorrhea are more geographically concentrated; these differential distributions may explain chlamydia’s lack of association with state-level minimum wages.

Findings should be considered in light of several limitations. Confined by ecological design, we analyzed only MSA-level data, so the effects of individual, risk-network and neighborhood-level factors are unknown. Due to CDC restrictions on STI data stratification we were unable to perform separate analyses for the sub-populations that are both most vulnerable to these STIs and more likely to earn minimum wages (young, black and/or Hispanic/Latina women) [68, 69]. This limitation might have changed the magnitude of the associations we found here. We note, however, the possibility that minimum wage policies may influence STI rates *across* the socioeconomic groups, when dissortative sexual networks connect affluent low STI-risk individuals with minimum wage-dependent high STI-risk individuals [14]. We did not account for city-level minimum wages, which might also have biased our results in unknown ways. Some covariates (e.g. teen births) may have been both confounders and mediators for the relationship between minimum wage and STIs, so we may have attenuated the effect by controlling for them [71]. Similarly, the relationship between minimum wage and employment may be recursive, so our preliminary mediation findings may also be subject to bias due to time-varying confounding. Our analysis spans the period of the Great Recession and subsequent economic recovery, as well as adoption of the Affordable Care Act, so the findings may be of limited generalizability to other periods. Undertesting and incomplete STI reporting may have introduced measurement error to our STI data, in particular to its earlier years [72].

This analysis has multiple strengths. We were able to analyze longitudinal data spanning thirteen years (2003–2015), making a stronger argument for causal inference as compared to cross-sectional or short-term studies. Our database also covered a significant portion (50.4%) of the US female population, enhancing the impact of our findings. We also had access to STI *diagnosis* data, rather than self-report data on STIs, avoiding social desirability and recall biases.

This is the first study of the relationship between minimum wage and common reportable non-HIV STIs. Future studies should focus on assessing the relationship between minimum wage laws and STIs in the most at-risk groups of women (i.e., young, black and/or Hispanic/Latina women); overcome the limitations of ecological design by using a multilevel design that included neighborhood-, network-, and individual-level-data; account for spatial and temporal variations in STI screening and treatment; and apply analytic strategies addressing time-varying confounding.

## Conclusions

Our findings suggest that increasing the minimum wage may have a protective effect on STI rates among women living in large MSAs in the US. Minimum wage debates in the US are mostly focused on the possible economic benefits of raising this wage. This study, combined with other emerging public health research [25–27], suggests that increasing the minimum wage also may improve the public’s health. If future research corroborates these findings, then minimum wage and other economic policies should become a part of comprehensive public health research and response strategy addressing STIs among economically deprived populations.

## Supporting information

S1 Table Data sources for rates and potential correlates of newly diagnosed cases of primary and secondary syphilis, gonorrhea, and chlamydia, among women per 100,000, residing in 68 large US metropolitan statistical areas s between 2003–2015. (DOCX)

S2 Table Descriptive statistics for potential mediators: 68 large US metropolitan statistical areas, 2002–2014. (DOCX)

S3 Table Results of hierarchical linear bivariate regressions of rates of primary and secondary syphilis, gonorrhea, and chlamydia among women per 100,000 over time and potential mediators, 68 large US metropolitan statistical areas, 2003–2015. (DOCX)

S4 Table Exploratory testing of select mediators of the relationship between state-level minimum wage and rates of primary and secondary syphilis and gonorrhea among women per 100,000: 66 large US metropolitan statistical areas, 2003–2015. (DOCX)
