# Supplementary material for: States with higher minimum wages have lower STI rates among women: Results of an ecological study of 66 US metropolitan areas, 2003-2015
Source: PLoS One. 2019 Oct 9;14(10):e0223579. doi: 10.1371/journal.pone.0223579 (PMC6785113; doi:10.1371/journal.pone.0223579)
Supplement: S1 Table — (DOCX) [file pone.0223579.s001.docx]

| **Supplemental table 1**. Data Sources for Rates and Potential Correlates of Newly Diagnosed Cases of Primary and Secondary Syphilis, Gonorrhea, and Chlamydia, among Women per 100,000, Residing in 66 Large US MSAs between 2003-2015^a^ | |
| --- | --- |
| **Variables** | **Data Source** |
| **Dependent Variable** | |
| Primary and secondary syphilis rates per 100,000 females | Centers for Disease Control and Prevention (2003-2015)^b^ |
| Gonorrhea rates per 100,000 females | Centers for Disease Control and Prevention (2003-2015)^b^ |
| Chlamydia rates per 100,000 females | Centers for Disease Control and Prevention (2003-2015)^b^ |
| **Independent Variable** | |
| Cost of living and inflation adjusted minimum wage – yearly average of monthly minimum wage | Monthly minimum wage and consumer price index for inflation adjustment - Komro et al (2002-2014)^c^  Cost of living index (2000)^d^ |
| **Potential Covariates** | |
| **Percent living in a health provider shortage area** | |
| Population 15-64 living in a health provider shortage area (numerator) | Health Resources and Services Administration  HRSA (2002)^e^ |
| Total MSA population of all age, race and gender ages 15-64 (denominator) | Intercensal Population Estimates – Census Bureau (2002)^f^ |
| **Syringes exchanged per injection drug users** | |
| No. syringes distributed (numerator) | Beth Israel National Survey of Syringe Exchange Programs/North American Syringe Exchange Network (2002)^g^ |
| No. injection drug users (denominator) | Tempalski et al.(2002)^h^ |
| **Black isolation index^i, j^** | Decennial Census – Census Bureau (2000)^k^  American Community Survey, Annual Social Economic Supplement – Census Bureau  (2008-2012 and 2012-2016, 5 year estimates)^l^ |
| **% residents 15-29** | |
| Total population ages 15-29 of all race and gender (numerator) | Intercensal Population Estimates – Census Bureau (2002-2014)^f^ |
| Total population of all age, race and gender (denominator) | Intercensal Population Estimates – Census Bureau (2002-2014)^f^ |
| **% Hispanic residents** | |
| Total Hispanic residents of all age and gender (numerator) | Intercensal Population Estimates – Census Bureau (2002-2014)^f^ |
| Total residents of all age and gender (denominator) | Intercensal Population Estimates – Census Bureau (2002-2014)^f^ |
| **% non-Hispanic black residents** | |
| Total non-Hispanic black residents of all age and gender (numerator) | Intercensal Population Estimates – Census Bureau (2002-2014)^f^ |
| Total residents of all age and gender (denominator) | Intercensal Population Estimates – Census Bureau (2002-2014)^f^ |
| **Population density^j^** | |
| Total MSA population of all age, race and gender (numerator) | Decennial Census – Census Bureau (2000, 2010)^k^ |
| Total MSA land area in sq. miles (denominator) | Decennial Census – Census Bureau (2000, 2010)^k^ |
| **% female-headed households^i, j^** | |
| No. female-headed households (numerator) | Decennial Census – Census Bureau (2000)^k^  American Community Survey, Annual Social Economic Supplement – Census Bureau (2008-2012 and 2012-2016, 5 year estimates)^l^ |
| No. households (denominator) | Decennial Census – Census Bureau (2000)^k^  American Community Survey, Annual Social Economic Supplement – Census Bureau  (2008-2012 and 2012-2016, 5 year estimates)^l^ |
| **Teen birth rate^j^** | |
| Births per 1,000 females aged 10-19 | CDC National Vital Statistics (2002-2013)^m^ |
| Total female population aged 10-19 | Intercensal Population Estimates (2002-2013)^f^ |
| **Male to female sex ratio for Hispanic adults^j^** | |
| No. Hispanic males ages 18-64 (numerator) | Decennial Census – Census Bureau (2000, 2010)^k^ |
| No. Hispanic females ages 18-64 (denominator) | Decennial Census – Census Bureau (2000, 2010)^k^ |
| **Male to female sex ratio for non-Hispanic black adults^j^** | |
| No. non-Hispanic black males ages 18-64 (numerator) | Decennial Census – Census Bureau (2000, 2010)^k^ |
| No. non-Hispanic black females ages 18-64 (denominator) | Decennial Census – Census Bureau (2000, 2010)^k^ |
| **% uninsured residents^j^** | |
| No. uninsured residents (numerator) | Small Area Health Insurance Estimates - Census Bureau (2005-2014)^n,o^ |
| No. total population (denominator) | Intercensal Population Estimates - Census Bureau (2005-2014)^f^ |
| **% uninsured women^j^** | |
| No. uninsured residents (numerator) | Small Area Health Insurance Estimates - Census Bureau (2005-2014)^n,o^ |
| No. total population (denominator) | Intercensal Population Estimates - Census Bureau (2005-2014)^f^ |
| **Cost of living and inflation adjusted health expenditures per capita^j^** | |
| Health expenditures (numerator) | US Census of Governments (2002, 2007, 2012)^p, q^ |
| No. total population (denominator) | Intercensal Population Estimates – Census Bureau (2002, 2007, 2012)^f^ |
| Consumer price index and cost of living index for adjustment | Consumer price index for inflation adjustment - Komro et al (2014)^c^  Cost of living index (2000)^d^ |
| **Cost of living and inflation adjusted community and housing development expenditures per capita^j^** | |
| Community and housing development expenditures (numerator) | US Census of Governments (2002, 2007, 2012)^p, r^ |
| No. total population (denominator) | Intercensal Population Estimates – Census Bureau (2002, 2007, 2012)^f^ |
| Consumer price index and cost of living index for adjustment | Consumer price index for inflation adjustment - Komro et al (2014)^c^  Cost of living index (2000)^d^ |
| **State Earned Income Tax Credit (EITC)** |  |
| State EITC rate for single women with one child as a percentage of federal EITC rate | Markowitz et al. (2017)^s^ |
| State EITC refund policy (yes/no) |  |
| **State % of SNAP recipients** | |
| No. SNAP recipients (numerator) | University of Kentucky Center for Poverty Research (2002-2014) |
| Population (denominator) |  |
| **State % of TANF recipients** | |
| No TANF recipients (numerator) | University of Kentucky Center for Poverty Research (2002-2014) |
| Population (denominator) |  |
| **Potential mediators** | |
| **% female adults with no high school diploma or equivalent^i,j^** | |
| No. female adults (25 and up) with no high school diploma or GED equivalent (numerator) | Decennial Census – Census Bureau (2000)^k^  American Community Survey, Annual Social Economic Supplement – Census Bureau  (2005-2009, 2006-2010, 2007-2011, 2008-2012, 2009-2013, 2010 -2014, 2011-2015, 2012-2016, 5 year estimates)^l^ |
| No. female adults for whom educational attainment status is determined (denominator) | Decennial Census – Census Bureau (2000)^k^  American Community Survey, Annual Social Economic Supplement – Census Bureau  (2005-2009, 2006-2010, 2007-2011, 2008-2012, 2009-2013, 2010 -2014, 2011-2015, 2012-2016, 5 year estimates)^l^ |
| **% male and female adults with no high school diploma or equivalent^i,j^** | |
| No. adults (25 and up) with no high school diploma or GED equivalent (numerator) | Decennial Census – Census Bureau (2000)^k^  American Community Survey, Annual Social Economic Supplement – Census Bureau  (2005-2009, 2006-2010, 2007-2011, 2008-2012, 2009-2013, 2010 -2014, 2011-2015, 2012-2016, 5 year estimates)^l^ |
| No. adults for whom educational attainment status is determined (denominator) | Decennial Census – Census Bureau (2000)^k^  American Community Survey, Annual Social Economic Supplement – Census Bureau  (2005-2009, 2006-2010, 2007-2011, 2008-2012, 2009-2013, 2010 -2014, 2011-2015, 2012-2016, 5 year estimates)^l^ |
| **% employed females^i, j^** | |
| No. females employed  (numerator) | Decennial Census – Census Bureau (2000)^k^  American Community Survey, Annual Social Economic Supplement – US Census Bureau (2005-2009, 2006-2010, 2007-2011, 2008-2012, 2009-2013, 2010 -2014, 2011-2015, 2012-2016, 5 year estimates)^l^ |
| No. female adults in the labor force and non-labor force (denominator) | Decennial Census – Census Bureau (2000)^k^  American Community Survey, Annual Social Economic Supplement – US Census Bureau (2005-2009, 2006-2010, 2007-2011, 2008-2012, 2009-2013, 2010 -2014, 2011-2015, 2012-2016, 5 year estimates)^l^ |
| **% employed males and females^i, j^** | |
| No. adults employed  (numerator) | Decennial Census – Census Bureau (2000)^k^  American Community Survey, Annual Social Economic Supplement – US Census Bureau (2005-2009, 2006-2010, 2007-2011, 2008-2012, 2009-2013, 2010 -2014, 2011-2015, 2012-2016, 5 year estimates)^l^ |
| No. adults in the labor force and non-labor force (denominator) | Decennial Census – Census Bureau (2000)^k^  American Community Survey, Annual Social Economic Supplement – US Census Bureau (2005-2009, 2006-2010, 2007-2011, 2008-2012, 2009-2013, 2010 -2014, 2011-2015, 2012-2016, 5 year estimates)^l^ |
| **Gini index^i, j^** | Share of aggregate income from Decennial census – Census Bureaus, (2000)^k^ and American Community Survey, Annual Social Economic Supplement – Census Bureau  (2005-2009, 2006-2010, 2007-2011, 2008-2012, 2009-2013, 2010 -2014, 2011-2015, 2012-2016, 5 year estimates)^l^ |
| **% female-headed households with income below poverty level^i, j^** | |
| No. female-headed households with income below poverty level (numerator) | Decennial Census – Census Bureau (2000)^k^  American Community Survey, Annual Social Economic Supplement – Census Bureau  (2005-2009, 2006-2010, 2007-2011, 2008-2012, 2009-2013, 2010 -2014, 2011-2015, 2012-2016, 5 year estimates)^l^ |
| No. female-headed households for whom poverty status is determined (denominator) | Decennial Census – Census Bureau (2000)^k^  American Community Survey, Annual Social Economic Supplement – Census Bureau  (2005-2009, 2006-2010, 2007-2011, 2008-2012, 2009-2013, 2010 -2014, 2011-2015, 2012-2016, 5 year estimates)^l^ |
| **% individuals with income below poverty level^i, j^** | |
| No. individuals (all ages) with income below poverty level (numerator) | Decennial Census – Census Bureau (2000)^k^  American Community Survey, Annual Social Economic Supplement – Census Bureau  (2005-2009, 2006-2010, 2007-2011, 2008-2012, 2009-2013, 2010 -2014, 2011-2015, 2012-2016, 5 year estimates)^l^ |
| No. individuals (all ages) for whom poverty status is determined (denominator) | Decennial Census – Census Bureau (2000)^k^  American Community Survey, Annual Social Economic Supplement – Census Bureau  (2005-2009, 2006-2010, 2007-2011, 2008-2012, 2009-2013, 2010 -2014, 2011-2015, 2012-2016, 5 year estimates)^l^ |
| **% incarcerated males^j^** | |
| No. males in correctional facilities (numerator) | Decennial Census – Census Bureau (2000, 2010)^k^ |
| No. adult males (denominator) | Decennial Census – Census Bureau (2000, 2010)k |
| **% incarcerated females^j^** | |
| No. females in correctional facilities (numerator) | Decennial Census – Census Bureau (2000, 2010)^k^ |
| No. adult females (denominator) | Decennial Census – Census Bureau (2000, 2010)k |
| **Low income households with rent > 30% of income^i, j^** | |
| No. households with income <%10,000 and with gross rent >30% of total household income (numerator) | Decennial Census – Census Bureau (2000)^k^  American Community Survey, Annual Social Economic Supplement – Census Bureau  (2005-2009, 2006-2010, 2007-2011, 2008-2012, 2009-2013, 2010 -2014, 2011-2015, 2012-2016, 5 year estimates)^l^ |
| Total number of renter-occupied units with household income <$10,000 (denominator) | Decennial Census – Census Bureau (2000)^k^  American Community Survey, Annual Social Economic Supplement – Census Bureau  (2005-2009, 2006-2010, 2007-2011, 2008-2012, 2009-2013, 2010 -2014, 2011-2015, 2012-2016, 5 year estimates)^l^ |

^a^ 2003 to 2015 refers to the timeframe for STI outcomes. Correlates were lagged 1 year and reflect 2002 to 2014*.*

^b^ Centers for Disease Control. Data received by request from the CDC, Nationally Notifiable Disease Surveillance System, Division of STD Prevention on November 22, 2017.

^c^ Komro KA, Livingston MD, Markowitz S, et al. The effect of an increased minimum wage on infant mortality and birth weight. American journal of public health 2016;106(8):1514-16.

^d^ Council for Community and Economic Research. Cost of living index. 2000. http://coli.org

^e^ Health Resources and Services Administration, Health Professional Shortage Area Website: <http://www.hrsa.gov/shortage/>. 2015.

^f^ Intercensal estimates of the resident population by sex, race, and Hispanic origin for counties. April 1, 2002 to July 1, 2014. Intercensal Estimates of the Resident Population. Washington, DC: US Census Bureau; 2015.

^g^ Syringe exchange coverage. 2007. Beth Israel National Survey of Syringe Exchange Programs. New York, New York: Beth Israel Medical Center; 2013.

^h^ Tempalski B, Pouget ER, Cleland CM, et al. Trends in the population prevalence of people who inject drugs in US metropolitan areas 1992–2007. PLoS One. 2013;8(6): e64789.

^i^ For these variables, the numerator and/or denominator across years were obtained from different census sources, the 2000 decennial census and the American Community Survey 5 year estimates. The 2000 census was a long form census and the 2010 census was a short form census. The 2010 short form census does not contain all the measures collected in the 2000 long form census; the American Community Survey (ACS) was implemented to collect this information with the first five-year estimate in 2005-2009. The midpoint of American Community Survey 5-year estimates is used for estimates after 2000.

^j^ Where yearly data were not available, missing year’s data were linearly interpolated between available year’s data or extrapolated based on 5-year average change for adjoining years.

^k^ US Census Bureau. Decennial Census 2000 and 2010. Washington, DC: US Census Bureau; American Fact Finder. 2000, 2010.

^l^ US Census Bureau. American Community Survey, Annual Social Economic Supplement Data, 2005-2009, 2006-2010, 2007-2011, 2008-2012, 2009-2013, 2010 -2014, 2011-2015, 2012-2016. Washington, DC: US Census Bureau; American Fact Finder.

^m^ National Vital Statistics System, <http://www.cdc.gov/nchs/births.htm> requested via National Association for Public Health Statistics and Information Systems (NAPHSIS) <http://www.naphsis.org/>, 2002-2013.

^n^ US Census Bureau. American Community Survey, Small Area Health Insurance Estimates; Washington, DC: US Census Bureau; 2015.

^o^ Health uninsurance rates are based on census, tax return, and insurance participation records as extracted from the US Census Small Area Health Insurance Estimates files.

^p^ US Census of Governments. County area finances file, 2012. Washington, DC: US Census Bureau; 2012.

^q^ Government health expenditures are defined as health operations, construction, and capital outlay expenditures for municipalities as extracted from the US Census County Area Finances File.

^r^ Government housing and community development expenditures are defined as housing and community development operations, construction, and capital outlay expenditures for municipalities as extracted from the US Census County Area Finances File.

^s^ Markowitz S, Komro KA, Livingston MD, Lenhart O, Wagenaar AC. Effects of state-level earned income tax credit laws in the US on maternal health behaviors and infant health outcomes. *Soc Sci Med*. 2017;194:67-75.

^t^ University of Kentucky Center for Poverty Research. 2019. UKCPR National Welfare Data, 1980-2017. URL: http://ukcpr.org/resources/national-welfare-data (Accessed June, 6, 2019).
