# Supplementary material for: States with higher minimum wages have lower STI rates among women: Results of an ecological study of 66 US metropolitan areas, 2003-2015
Source: PLoS One. 2019 Oct 9;14(10):e0223579. doi: 10.1371/journal.pone.0223579 (PMC6785113; doi:10.1371/journal.pone.0223579)
Supplement: S2 Table — (DOCX) [file pone.0223579.s002.docx]

| **Supplemental Table 2. Descriptive Statistics for Potential Mediators: 66 Large US Metropolitan Statistical Areas, 2002 – 2014^a^** | | | | | |
| --- | --- | --- | --- | --- | --- |
| Potential Mediators | Mean | Std Dev | Median | 25th Pctl | 75th  Pctl |
| % of female-headed households with income below poverty level |  |  |  |  |  |
| *Lagged baseline (2002)* | 4.36 | 1.25 | 4.18 | 3.52 | 5.06 |
| *Change between 2002 and 2014* | 0.84 | 0.62 | 0.81 | 0.47 | 1.27 |
| % of individuals with income below poverty level |  |  |  |  |  |
| *Lagged baseline (2002)* | 11.85 | 4.49 | 11.02 | 9.52 | 12.62 |
| *Change between 2002 and 2014* | 2.61 | 1.40 | 2.62 | 1.83 | 3.54 |
| Gini index |  |  |  |  |  |
| *Lagged baseline (2002)* | 0.45 | 0.02 | 0.44 | 0.43 | 0.45 |
| *Change between 2002 and 2014* | 0.02 | 0.01 | 0.02 | 0.01 | 0.02 |
| % of employed adults (aged 16-64) |  |  |  |  |  |
| *Lagged baseline (2002)* | 60.81 | 4.79 | 61.35 | 58.55 | 63.96 |
| *Change between 2002 and 2014* | -1.21 | 1.93 | -1.43 | -2.48 | -0.31 |
| % of employed females (aged 16-64) |  |  |  |  |  |
| *Lagged baseline (2002)* | 55.25 | 4.82 | 55.76 | 53.49 | 58.20 |
| *Change between 2002 and 2014* | 2.10 | 1.79 | 1.90 | 1.01 | 3.17 |
| % of adults (25 and up) without a high school diploma or equivalent |  |  |  |  |  |
| *Lagged baseline (2002)* | 17.13 | 4.98 | 15.81 | 14.38 | 18.08 |
| *Change between 2002 and 2014* | -5.00 | 1.70 | -4.86 | -5.97 | -3.82 |
| % of female adults (25 and up) without a high school diploma or equivalent |  |  |  |  |  |
| *Lagged baseline (2002)* | 16.81 | 4.69 | 15.85 | 14.23 | 17.48 |
| *Change between 2002 and 2014* | -5.31 | 1.66 | -5.13 | -6.47 | -4.00 |
| % incarcerated males |  |  |  |  |  |
| *Lagged baseline (2002)* | 1.54 | 0.69 | 1.43 | 1.01 | 1.94 |
| *Change between 2002 and 2014* | -0.07 | 0.40 | -0.10 | -0.23 | 0.13 |
| % incarcerated females |  |  |  |  |  |
| *Lagged baseline (2002)* | 0.22 | 0.32 | 0.14 | 0.09 | 0.24 |
| *Change between 2002 and 2014* | -0.02 | 0.14 | -0.00 | -0.05 | 0.04 |
| Low income households with rent > 30% of income |  |  |  |  |  |
| *Lagged baseline (2002)* | 70.25 | 3.32 | 69.62 | 68.07 | 72.12 |
| *Change between 2002 and 2014* | -1.52 | 3.69 | -0.97 | -3.88 | 0.78 |

^a^ 2003 to 2015 is the timeframe for the STI outcome. Correlates were lagged 1 year and reflect 2002-2014. Descriptive statistics were assessed for 66 MSAs with available data on exposure (price-adjusted minimum wage).

Note: Correlates were lagged one year because we did not expect a change in the correlates to have an instantaneous effect on the outcome.
