# Supplementary material for: States with higher minimum wages have lower STI rates among women: Results of an ecological study of 66 US metropolitan areas, 2003-2015
Source: PLoS One. 2019 Oct 9;14(10):e0223579. doi: 10.1371/journal.pone.0223579 (PMC6785113; doi:10.1371/journal.pone.0223579)
Supplement: S3 Table — (DOCX) [file pone.0223579.s003.docx]

| **Supplemental table 3. Results of Hierarchical Linear Bivariate Regressions of Rates of Primary and Secondary Syphilis, Gonorrhea, and Chlamydia Among Women per 100,000 Over Time and Potential Mediators, 66 Large US Metropolitan Statistical Areas, 2003-2015^a^** | | | |
| --- | --- | --- | --- |
| Potential Mediators | Log syphilis  bivariate models^b^  standardized coefficient  (95% CI) | Gonorrhea  bivariate models^b^  standardized  coefficient  (95% CI) | Chlamydia  bivariate models^b^  standardized  coefficient  (95% CI) |
| % of female-headed households with income below poverty level |  |  |  |
| *Lagged baseline (2002)* | 0.22 (0.04, 0.39) | 0.38 (0.17, 0.58) | 0.56 (0.38, 0.74) |
| *Change since 2002* | -0.05 (-0.17, 0.08) | 0.12 (0.03, 0.20) | -0.01 (-0.09, 0.07) |
| % of individuals with income below poverty level |  |  |  |
| *Lagged baseline (2002)* | 0.11 (-0.08, 0.30) | 0.10 (-0.13, 0.33) | 0.48 (0.16, 0.78) |
| *Change between 2002 and 2014* | -0.09 (-0.24, 0.06) | 0.05 (-0.05, 0.14) | -0.09 (-0.17, 0.00) |
| Gini index |  |  |  |
| *Lagged baseline (2002)* | 0.09 (-0.10, 0.29) | -0.03 (-0.27, 0.20) | -0.01 (-0.26, 0.24) |
| *Change since 2002* | -0.05 (-0.2, 0.11) | 0.00 (-0.10, 0.10) | -0.05 (-0.14, 0.03) |
| % of employed adults (aged 16-64) |  |  |  |
| *Lagged baseline (2002)* | 0.07 (-0.15, 0.3) | 0.12 (-0.15, 0.38) | 0.01 (-0.25, 0.27) |
| *Change between 2002 and 2014* | 0.13 (0.00, 0.27) | -0.01 (-0.10, 0.08) | 0.12 (0.04, 0.19) |
| % of employed females (aged 16-64) |  |  |  |
| *Lagged baseline (2002)* | 0.04 (-0.18, 0.27) | 0.12 (-0.15, 0.38) | 0.07 (-0.18, 0.31) |
| *Change since 2002* | 0.02 (-0.08, 0.13)* | -0.12 (-0.18, -0.06)* | 0.05 (-0.00, 0.10) |
| % of adults (25 and up) without a high school diploma or equivalent |  |  |  |
| *Lagged baseline (2002)* | 0.06 (-0.18, 0.29) | -0.03 (-0.30, 0.24) | 0.17 (-0.08, 0.43) |
| *Change between 2002 and 2014* | -0.11 (-0.36, 0.15) | -0.07 (-0.24, 0.11) | -0.18 (-0.33, -0.03) |
| % of female adults (25 and up) without a high school diploma or equivalent |  |  |  |
| *Lagged baseline (2002)* | 0.02 (-0.20, 0.25) | -0.06 (-0.32, 0.20) | 0.17 (-0.08 0.42) |
| *Change since 2002* | -0.12 (-0.38, 0.13) | -0.11 (-0.28, 0.06) | -0.16 (-0.31, -0.01) |
| % incarcerated males |  |  |  |
| *Lagged baseline (2002)* | 0.00 (-0.17, 0.18) | 0.09 (-0.13, 0.31) | 0.21 (-0.02, 0.44) |
| *Change since 2002* | -0.07 (-0.19, 0.06) | 0.12 (0.03, 0.21) | 0.00 (-0.08, 0.09) |
| % incarcerated females |  |  |  |
| *Lagged baseline (2002)* | 0.03 (-0.15, 0.21) | 0.05 (-0.17, 0.27) | 0.22 (-0.02, 0.46) |
| *Change since 2002* | 0.03 (-0.10, 0.16) | 0.01 (-0.08, 0.11) | -0.05 (-0.13, 0.03) |
| Low income households with rent > 30% of income |  |  |  |
| *Lagged baseline (2002)* | -0.07 (-0.28, 0.15) | -0.03 (-0.28, 0.23) | 0.13 (-0.11, 0.36) |
| *Change since 2002* | 0.02 (-0.08, 0.12) | -0.02 (-0.09, 0.05) | -0.00 (-0.06, 0.06) |

^a^ 2003 to 2015 is the timeframe for the STI outcome. Bivariate models were assessed for 68 MSAs with available data on exposure (price-adjusted minimum wage). Potential mediators were lagged 1 year and reflect 2002-2014 because we did not expect a change in the potential mediators to have an instantaneous effect on the outcome.

^b^ The bivariate models include time, state as a random effect, and standardized potential mediator dyad (baseline and change values).

*Bivariate models where the inclusion of the correlate dyad changed the magnitude of the association between the focal exposure variable (baseline minimum wage and/or change since 2002) and the STI outcome by 10% or more.
