# Supplementary material for: States with higher minimum wages have lower STI rates among women: Results of an ecological study of 66 US metropolitan areas, 2003-2015
Source: PLoS One. 2019 Oct 9;14(10):e0223579. doi: 10.1371/journal.pone.0223579 (PMC6785113; doi:10.1371/journal.pone.0223579)
Supplement: S4 Table — (DOCX) [file pone.0223579.s004.docx]

| **Supplemental Table 4. Exploratory Testing of Select Mediators of the Relationship Between State-Level Minimum Wage and Rates of Primary and Secondary Syphilis and Gonorrhea among Women per 100,000: 66 Large US Metropolitan Statistical Areas, 2003-2015** | | | | |
| --- | --- | --- | --- | --- |
| **Potential mediators^b^** | **Log syphilis**  **multivariate model^c^**  **standardized coefficient**  **(95% CI)** | **% change^d^ in the standardized coefficients for the focal exposure^e^ for syphilis model** | **Gonorrhea**  **multivariate**  **model^c^**  **standardized**  **coefficient**  **(95% CI)** | **% change^d^ in the standardized coefficients for the focal exposure^e^ for gonorrhea model** |
| % of employed females (aged 15-64) |  |  |  |  |
| *Lagged baseline (2002)* | 0.04 (-0.18, 0.25) |  | 0.00 (-0.20, 0.20) |  |
| *Change since 2002* | 0.01 (-0.10, 0.12) | -1.34% | -0.10 (-0.17, -0.03) | -21.96% |
| Note:  ^a^ 2003 to 2015 is the timeframe for the STI outcome. Covariates were lagged 1 year and reflect 2002-2014 because we did not expect a change in the covariates to have an instantaneous effect on the outcome.  ^b^ Covariates that passed mediation test (changing the magnitude of standardized coefficients for minimum wage > 10%) in the bivariate modelling stage  ^c^ Multivariate models include only covariates that changed the magnitude of association between the focal exposure dyad (baseline minimum wage and change since 2002) and an STI by 10% or more in the bivariate modelling stage (see Tables 2 and 3).  ^d^ Compared to the sum of the absolute values of standardized coefficients of the focal exposure dyad (baseline minimum wage and change in minimum wage since baseline) for the final model without mediators (see Table 3).  ^e^ Refers to change over time in state-level adjusted minimum wage only, since baseline minimum wage was not significantly associated with STI rates | | | | |
